# Perioperative onset of acquired von Willebrand syndrome: Comparison between HVAD, HeartMate II and on-pump coronary bypass surgery

**DOI:** 10.1371/journal.pone.0171029

**Published:** 2017-02-24

**Authors:** Christina Feldmann, Rashad Zayat, Andreas Goetzenich, Ali Aljalloud, Eva Woelke, Judith Maas, Lachmandath Tewarie, Thomas Schmitz-Rode, Ruediger Autschbach, Ulrich Steinseifer, Ajay Moza

**Affiliations:** 1 Department of Cardiovascular Engineering, Institute of Applied Medical Engineering, Helmholtz Institute, RWTH Aachen University, Aachen, Germany; 2 Department of Thoracic and Cardiovascular Surgery, University Hospital RWTH Aachen, Aachen, Germany; Virginia Commonwealth University Medical Center, UNITED STATES

## Abstract

**Objectives:**

Acquired von Willebrand syndrome (AvWS) is associated with postoperative bleeding complications in patients with continuous flow left ventricular assist devices (CF-LVADs). The aim of this study is to analyze the perioperative vWF profile comparing an axial pump (HMII) to a centrifugal pump (HVAD) regarding the correlation between perioperative occurrence of AvWS, early- and late-postoperative bleeding events.

**Methods:**

From July 2013 until March 2015 blood samples of 33 patients (12 HMII/ 8 HVAD/ 13 controls) were prospectively collected at 12 different time points and analyzed for the vWF antigen (vWF:Ag), its activity (vWF:Ac) and the vWF:Ac/vWF:Ag-ratio (vWF:ratio). The follow up period for postoperative bleeding events was from July 2013 until July 2016.

**Results:**

Postoperatively, there was no difference in the vWF-profile between HVAD and HMII groups. However, a subgroup of patients already had significantly lower vWF:ratios preoperatively. Postoperatively, both CF-LVAD groups presented significantly lower vWF:ratios compared to the control group. Bleeding events per patient-year did not differ between the two groups (HMII vs. HVAD: 0.67 vs. 0.85, *p* = 0.685). We detected a correlation between vWF:ratio <0.7at LVAD-start (*r* = -0.583, *p* = 0.006) or at the end of surgery (*r* = -0.461, *p* = 0.035) and the occurrence of pericardial tamponade. In the control group, the drop in both vWF:Ag and vWF:Ac recovered immediately postoperatively above preoperative values.

**Conclusion:**

A subgroup of patients with end-stage heart failure already suffers AvWS preoperatively. In both CF-LVAD groups, AvWS begins immediately after surgery. Intraoperative vWF:ratios <0.7 correlate with higher incidences of pericardial tamponade and re-operation. The presumably dilutive effect of the heart lung machine on vWF vanishes immediately at the end of surgery, possibly as part of an acute-phase response.

## Introduction

Continuous-flow left ventricular assist devices (CF-LVADs) have gained broad acceptance in the therapy of end-stage heart failure as either a bridge to transplantation or destination therapy [1, 2]. With an incidence between 18% and 40%, thromboembolic and bleeding events, especially gastrointestinal (GI) bleeding, still present a major postoperative complication [3]. Several factors contribute to the high incidence of bleeding after CF-LVAD implantation. Among the most discussed factors is the acquired von Willebrand syndrome (AvWS) as a result of mechanical destruction and proteolysis of high-molecular-weight multimers (HMWM) of von Willebrand factor (vWF), induced by shear stress [4–6]. Previous studies investigated the time-course and onset of AvWS before/after CF-LVAD implantation and its correlation to bleeding events. It has been proven that virtually all patients develop AvWS after CF-LVAD implantation [7–9]. Timewise, analysis and comparison were only made between preoperative values of vWF and the first postoperative day [10].

There remains a lack of information about the perioperative changes in vWF profile, the onset of AvWS in CF-LVADs patients and its correlation to early postoperative bleeding events.

## Materials and methods

We prospectively investigated the perioperative vWF profile in patients undergoing CF-LVAD implantation, comparing an axial pump (HeartMate II (HMII): St. Jude Medical, Minneapolis, USA) to a centrifugal pump (HVAD: HeartWare, Inc., Framingham, Massachusetts, USA) regarding the correlation between perioperative occurrence of AvWS and early-postoperative bleeding events.

As control-group, we analyzed changes in the vWF profile in patients undergoing routine coronary artery bypass grafting (CABG) surgery with heart lung machine (HLM) (CABG group).

### Patients and data collection

**Fig 1:**
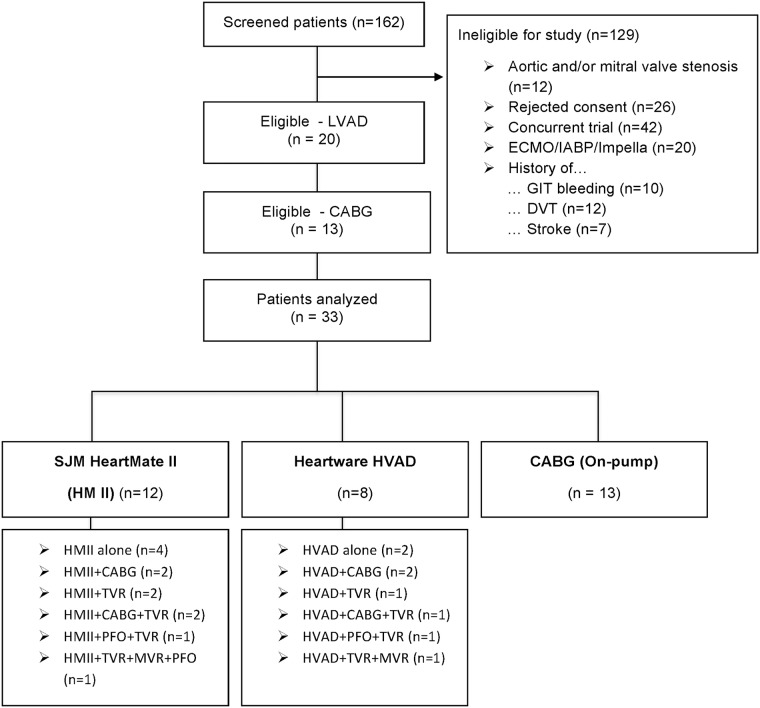
Study design and groups characteristics. CABG: coronary artery bypass graft; DVT: deep vein thrombosis; ECMO: extracorporeal membrane oxygenation; GI bleeding: gastrointestinal tract bleeding; HMII: HeartMate II; MVR: mitral valve reconstruction; IABP: intra-artery balloon pump; LVAD: left ventricular assist device PFO: patent foramen ovale; TVR: tricuspid valve reconstruction.

This was a prospective single-center cohort study. After approval by the local ethic committee (Ethik Komission an der RWTH, Aachen, Germany, EKK 150/09), 162 patients were screened from September 2013 to July 2015. All patients who were planned to receive a CF-LVAD implantation during the mentioned period were screened for this study. The exclusion criteria were: 1) preoperative need of extracorporeal life support (ECLS), 2) preoperative use of intra-aortic balloon pump (IABP), 3) preoperative use of Impella^®^, 4) moderate to severe aortic or mitral valve stenosis, 5) bleeding and/or 6) history of thromboembolic events (Fig 1).

Twenty CF-LVAD patients were eligible for the study. Informed written consent was obtained from each patient. 12 patients had HMII implantation (HMII group) (mean age 60.1±7.3 yrs, 1 female), 8 patients received an HVAD (HVAD group) (mean age 67±6.4 yrs, 3 females). 13 patients undergoing CABG as on-pump surgery (CABG group; mean age 67.6±8.4 yrs, 5 females) were recruited as control patients. Demographic characteristics, medical history, blood test values, medications, peri-, postoperative data and amount of transfusions were prospectively collected and stored in the clinical electronic database. European System for Cardiac Operative Risk Evaluation II (EuroSCORE II) was calculated for all patients and INTERMACS levels were determined for CF-LVADs patients. All CF-LVAD patients were routinely followed-up in our outpatient department. Last patient was included in March 2015 and the follow-up period was from July 2013 until July 2016.

### Bleeding events

Each patient’s bleeding history was evaluated during the stationary treatment and at every outpatient visit from July 2013 until July 2016. Bleeding types were defined as followed: 1) Intrathoracic bleedings (hematothorax or pericardial tamponade): decreased hemoglobin level, the need of transfusions and re-exploration; 2) GI bleeding: presence of melena, decreased hemoglobin level, required transfusions and endoscopic confirmation; 3) Epistaxis: nasal bleeding requiring medical intervention; 4) Intracerebral bleedings: onset of neurological dysfunction with confirmation through cerebral computer-tomography.

Bleeding events occurring during the first 30 days postoperative (POD) were considered early bleeding events.

### Anticoagulation regimen after implantation of CF-LVAD

Continuous infusion of heparin was initiated within a 12-hour postoperative period when the chest tube drainage was <50 mL/h, in order to achieve an activated partial thromboplastin time of 50–60 seconds. For platelet inhibition, acetylsalicylic acid (100 mg/d) was added to the medication on POD 1. At POD 3, phenprocoumon was given orally with a target international normalized ratio (INR) of 1.8–2.2 for HMII patients and 2.5–3.0 for HVAD patients.

### Blood collection and laboratory assays

Next to routine parameters, 12 pre-, peri- and postoperative blood samples were drawn for vWF assessment. Blood was collected in citrate containers (S-Monovette, 3,2% trisodium citrate/citric acid buffer solution, Sarstedt GmbH, Germany), processed to platelet poor plasma by centrifugation (10 min @ 8,000g) and stored at -80° until analyzed. vWF constitution was assessed via changes to the vWF antigen level (vWF:Ag, vWF Ag^®^ test kit, normal range 50–160%) and to the platelet binding activity level (vWF:Ac, INNOVANCE^®^ VWF Ac test kit normal range 47.8–173.2%), by turbidimetric analysis on a Sysmex CA 560 (test kits and device: Siemens Healthcare Diagnostic Products GmbH, Erlangen, Germany). Low specific activity (vWF:Ac/vWF:Ag ratio, in the following described as vWF:ratio for better readability) correlates with the loss of high-molecular-weight multimers (HMWM) and is therefore a measure for AvWS (vWF:ratio <0.7) [11]. For the CF-LVAD patients’ 2-months follow-up samples, analyses were derived from the clinical database and performed in the reference laboratory (MVZ labor, Dr. reising-Ackerman, Leipzig, Germany) as a routine diagnostic in our out-patient department using western blot technique for vWF multimer analysis and using enzyme-linked immunosorbent assay (ELISA) for vWF: Ristocetin Cofactor (vWF:RCo = vWF:Ac) and vWF:Ag measurements. Many previous studies did analyze a large sample of healthy individuals and patients with von Willebrand disease using these two different assays, the turbidimetric method (INNOVANCE^®^ vWF:Ac kit) and the ELISA method (vWF:RCo), finding a very good correlation between the two methods [12–14].

### Statistical analyses

Continuous variables are expressed as means ± standard deviation (SD) and categorical variables as absolute numbers and percentages. The comparison of demographics, pre- and postoperative data between the groups were performed with the two-tailed student’s t-test for continuous variables and Fisher’s exact test or χ2 test for categorical variables.

The comparisons of the repeated measured values of vWF:Ag, vWF:Ac and vWF:ratio, within each group and between the groups were carried out using the two-way ANOVA test with Tukey’s or Sidak’s test for correction of multiple comparisons, when appropriate.

All *p* values were then reported as adjusted *p* values to the count of comparisons. Bleeding rate was calculated as bleeding events per patient-years. Comparisons of the bleeding events per patient-years between groups were carried out with Cochran-Mantel-Haenszel test. For the correlation between vWF:ratio, bleeding events and transfused blood products the Pearson product moment correlation test was used. All statistical analyses were performed using SPSS software, version 23.0 (Chicago, IL, USA). An adjusted *p*-value of < 0.05 was considered significant. All *p*-values were reported at least as three digit numbers.

## Results and discussion

Demographics, pre-, peri-, postoperative data are listed in Tables 1 and 2. HVAD patients were older than HMII patients (67±6.4 yrs. vs. 60.1±7.3 yrs., *p* = 0.043) and had a lower body mass index (21.6±2.7 kg/m^2^ vs. 26.8±3.8 kg/m^2^, *p* = 0.002). Neither group, HMII or HVAD, differed in any other preoperative data and risk factors. Patients who received CF-LVADs implantation had a significantly lower left ventricular ejection fraction (LVAD vs. CABG: 19.1±3.3% vs. 50.3±6.4%, *p* = 0.0001) and a significantly higher EuroSCORE II than patients who underwent routine CABG surgery (LVAD vs. CABG: 8.6±4.2% vs. 2.5±1.4%, *p* = 0.0001).

**Table 1:** Demographic and perioperative data.

Variables	HVAD (n = 8)	HVAD vs. CABG	CABG (n = 13)	HMII vs. CABG	HM II (n = 12)	HVAD vs. HMII
**Age**	67 ± 6.4	0.731	67.6 ± 8.4	0.017	60.1 ± 7.3	0.043
**Female**	3 (37.5)	0.999	5 (38.5)	0.160	1 (8.3)	0.255
**BMI kg/m^2^**	21.6 ± 2.7	0.005	26.4 ± 4	0.800	26.8 ± 3.8	0.002
**ICM n (%)**	5 (62.5)	-	-	-	9 (75)	0.624
**DCM n (%)**	3 (37.5)	-	-	-	3 (25)	0.624
**LVEF %**	17.8 ± 2.1	0.0001	50.3 ± 6.4	0.0001	20.5 ± 4.5	0.132
**DMID n (%)**	2 (25)	0.399	6 (46.1)	0.999	5 (41.6)	0.655
**PAD n (%)**	2 (25)	0.655	5 (38.4)	0.168	1 (8.3)	0.563
**CVD n (%)**	1 (12.5)	0.606	4 (30.8)	0.321	1 (8.3)	1.000
**AF n (%)**	5 (62.2)	0.163	3 (23)	0.999	2 (16.6)	0.062
**Prior cardiac surgery n (%)**	2 (25)	-	0	-	3 (25)	0.999
**Prior PCI with stenting n (%)**	5 (62.2)	0.659	6 (46.2)	0.999	5 (41.6)	0.649
**EuroSCORE II %**	10.2 ± 5.7	0.0002	2.5 ± 1.4	0.0001	7.1 ± 2.7	0.117
**INTERMACS n (%):**						
**1**	1 (12.5)	-	-	-	1 (8.3)	1.000
**2**	1 (12.5)	-	-	-	2 (16.6)	0.999
**3**	5 (62.2)	-	-	-	6 (50)	0.669
**4**	1 (12.5)	-	-	-	3 (25)	0.618
**Blood Type O**	2 (25)	1.000	3 (23.1)	0.593	1 (8.3)	0.536
**Pre-Op data:**						
**VKA n (%)**	4 (50)	0.345	3 (23)	0.999	2 (16.6)	0.161
**ASA n (%)**	8 (100)	-	13(100)	-	12 (100)	1.000
**Clopidogrel**	0	-	6 (46.2)	-	0	-
**Hb g/dL**	11.6 ± 1.2	0.018	13.9 ± 1.4	0.150	12.7 ±2.4	0.136
**Platelets c/nL**	237.9 ± 53.5	0.750	231.3 ± 60.7	0.072	287.8 ± 87.9	0.113
**Peri-op data:**						
**Bypass-time min.**	104 ± 38.6	0.606	117 ± 66.8	0.963	118 ± 32.6	0.362
**PRBCs**	1.8 ± 1.8	0.588	1.4 ± 1.5	0.028	2.9 ± 1.7	0.182
**FFPs**	1.1 ± 2.1	0.694	0.7 ± 2.3	0.007	3.2 ± 1.8	0.027
**PCs**	1.1 ± 0.9	0.044	0.4 ± 0.6	0.013	1.7 ± 1.2	0.244

BMI: Body Mass Index; ICM: Ischemic Cardiomyopathy; DCM: Dilative Cardiomyopathy; LVEF: Left Ventricular Ejection Fraction; DMID: Diabetes Mellitus Insulin Depended; PAD: Peripheral Arterial Disease; CVD: Cerebrovascular Disease; AF: Atrial Fibrillation; PCI: Percutaneous Intervention; EuroSCOREII: European System for Cardiac Operative Risk Evaluation II; INTERMACS: Interagency Registry for Mechanically Assisted Circulatory Support; VKA: Vitamin-K-Antagonist; ASA: Acetylsalicylic Acid; Hb: Hemoglobin; PRBCs: Packed Red Blood Cells; FFPs: Fresh Frozen Plasmas; PCs: Platelet Concentrates. Continuous variables are expressed as mean ± SD and categorical variables as absolute numbers and percentages.

**Table 2:** Postoperative data.

Variables	HVAD (n = 8)	HVAD vs. CABG	CABG (n = 13)	HMII vs. CABG	HM II (n = 12)	HVAD vs. HMII
						
**Pneumonia**	3 (37.5)	0.252	1 (7.7)	0.160	4 (33.3)	1.000
**Sepstic shock**	2 (25)	0.133	0	0.220	2 (16.6)	1.000
**ARF**	3 (37.5)	0.042	0	0.220	2 (16.6)	0.347
**PRBCs**	4.7±4.5	0.004	0.6±0.9	0.003	4.3±4.0	0.837
**FFPs**	1.7±3.9	-	0	-	1.5±1.9	0.879
**PCs**	0.5±1.5	0.335	0.09±0.2	0.387	0.2±0.4	0.513
**ICU stays hrs.**	234.7±202.2	0.013	67.7±80.5	0.014	289.3±292.3	0.652
**Hospital LOS d**	25±15	0.057	14±10	0.025	25±13	0.925
**30 days mortality**	2 (25)	0.133	0	0.488	1 (8.3)	0.563
**Post-op bleeding n (%)**	<30d/ >30d		<30d / >30d		<30d/ >30d	
**GI**	0 / 2 (25)	0.133	0 / 0	0.488	0 / 1 (8.3)	0.563
**Hemothorax**	1 (12.5)/0	1.000	1 (7.6) / 0	0.073	3 (25) / 0	0.321
**Pericardial tamponade**	3 (37.5)/0	0.042	0 / 0	0.481	1 (8.3)/0	0.255
**Epistaxis**	0 / 0	-	0 / 0	0.488	1 (8.3) / 0	1.000
**Intracerebral**	0 / 0	-	0 / 0	0.488	0 / 1 (8.3)	1.000

ARF; Acute Renal Failure; ICU: Intensive Care Unit; LOS: Length of Stay; GI: Gastrointestinal; for further abbreviation please refer to Table 1.

Concomitant procedures were performed in 8 patients of the HMII group (n = 12) and in 6 patients of the HVAD group (n = 8) (Fig 1). All patients survived surgery. In both CF-LVADs groups there was 100% survival during the first 30 POD. The median CF-LVAD support time for HMII patients was 512±277 days (range: 33 to 904 days) versus 462±377 days (range: 36 to 983 days) for the HVAD patients (*p* = 0.739). During the follow-up period, five patients in the HVAD group and two patients in the HMII group (*p* = 0.065) died. One patient in the HMII group suffered ischemic cerebrovascular accident. During the follow-up period, no device thrombus could be detected in either HVAD or HMII group.

### vWF profile within each group

**Fig 2:**
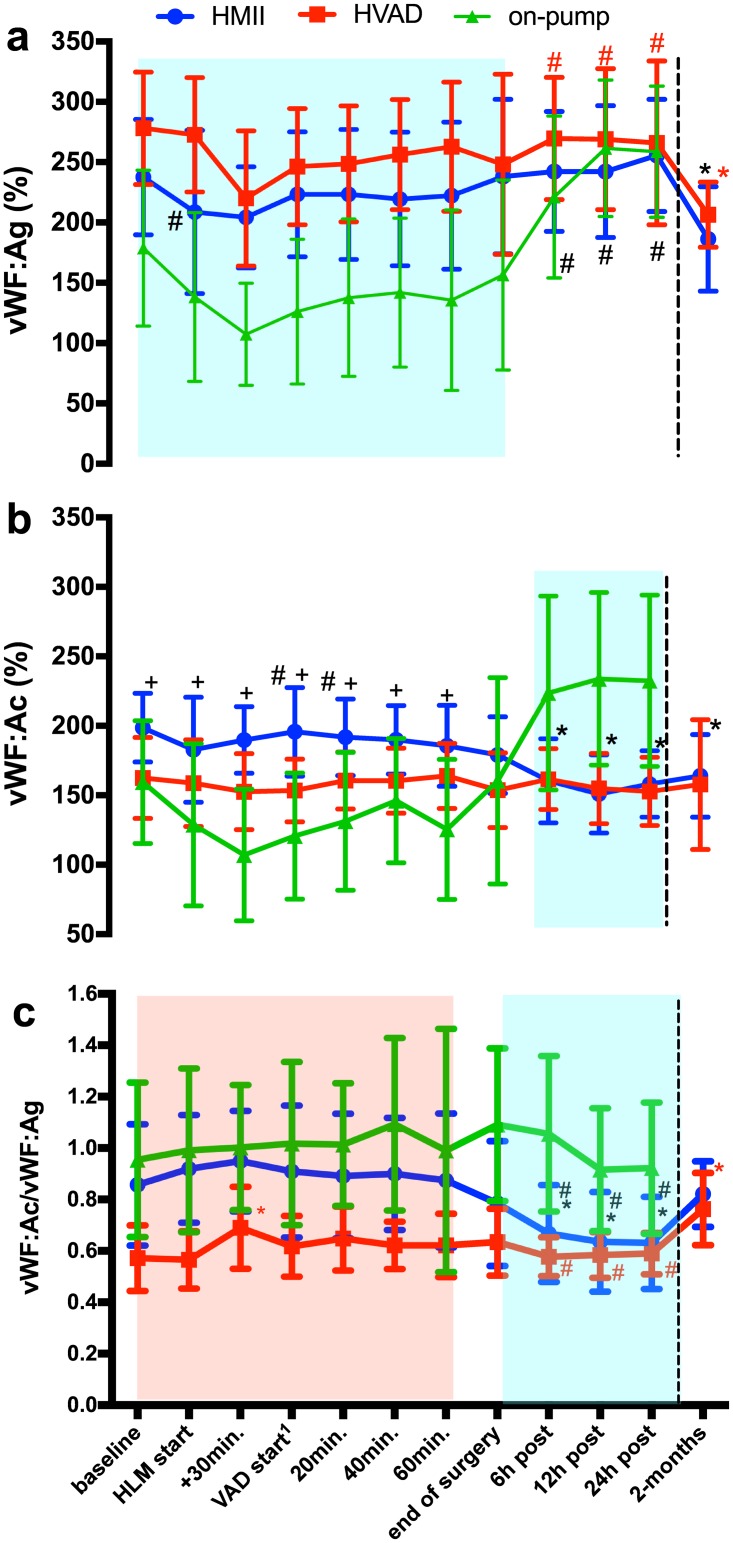
Time course of vWF:Ag, vWF:Ac and vWF:ratio in HMII, HVAD and CABG group. a) Time course of vWF:Ag, b) Time course of vWF:Ac, c) Time course of vWF:ratio. 1: Comparable time-points were chosen for the CABG control group, for more details please refer to Tables 3–5; Symbols: * indicates significance within group compared to baseline value; ^#^ indicates significant differences within groups compared to 2-month postop value (Black: HMII group, Red: HVAD group); ^+^ indicates significant differences between HMII and CABG; Areas: significant difference as compared to CABG (blue: both CF-LVAD groups; red: HVAD vs. both CABG and HM II). 2 months values were measured with ELISA as indicated by the dotted line; For further abbreviation please refer to Tables 3–5.

vWF:Ag and vWF:Ac levels and vWF:ratios are presented in Tables 3–5 and Fig 2.

**Table 3:** Von Willebrand factor antigen levels.

Time points LVAD groups/ CABG group	HVAD (n = 8)	*p*-values within HVAD group	HM II (n = 12)	*p*-values within HMII group	CABG (n = 13)	*p*-values within CABG group	*p*-values HM II vs. HVAD	*p*-values HMII vs. CABG	*p*-values HVAD vs. CABG
	vWF:Ag %	compared to baseline	vWF:Ag %	compared to baseline	vWF:Ag %	compared to baseline			
**Baseline**	278.1±46.6	-	237.5±47.8	-	178.8±64.6	-	0.315	0.034	0.001
**HLM start**	272.7±47.4	0.972	208.7±67.6	0.220	138.3±69.9	0.023	0.058	0.008	<0.0001
**30min**	220.1±56.1	0.001	204.3±41.9	0.0007	107.3±42.4	0.0001	0.840	0.0001	0.0002
**VAD start/60min**	246.3±48.1	0.117	223.4±51.7	0.593	126.1±59.9	0.0001	0.690	0.0001	<0.0001
**20min/90min**	248.6±47.9	0.515	223.2±53.8	0.603	137.7±65.2	0.001	0.636	0.0009	0.002
**40min/ 120min**	256.3±45.6	0.648	219.4±55.3	0.611	141.9±61.8	0.009	0.384	0.003	0.0001
**60min/ 150min**	262.8±53.5	0.724	222.3±60.9	0.901	135.6±74.8	0.016	0.315	0.0008	<0.0001
**End of OP**	248.2±74.7	0.674	238.1±63.9	0.999	156.5±78.8	0.526	0.930	0.0017	0.0027
**6h post**	269.5±50.6	0.994	242.3±49.7	0.999	221.2±67.1	0.112	0.592	0.640	0.185
**12h post**	269.1±58.5	0.993	242.2±54.5	0.999	261.5±56.6	0.0005	0.602	0.691	0.959
**24h post**	266.1±67.9	0.993	255.5±46.4	0.666	258.6±54.4	0.0006	0.925	0.991	0.612
**2-months**	206.4±26.9	0.006	186.3±43.3	0.012	-		0.998	-	-

CABG: Coronary artery bypass graft**;** End of OP: End of Operation; HLM: Heart Lung Machine; post: postoperative; VAD: Ventricular Assist Device; vWF:Ag: Von Willebrand Factor Antigen level. Results are expressed as means ± SD, *p*-values computed with the two-way-ANOVA test.

**Table 4:** Von Willebrand factor activity levels.

Time points LVAD groups/ CABG group	HVAD (n = 8)	*p*-values within HVAD group	HM II (n = 12)	*p*-values within HMII group	CABG (n = 13)	*p*-values within CABG group	*p*-values HMII vs. HVAD	*p*-values HMII vs. CABG	*p*-values HVAD vs. CABG
	vWF:Ac %	compared to baseline	vWF:Ac %	compared to baseline	vWF:Ac %	compared to baseline			
**Baseline**	162.5±29.2	-	198.6±24.7	-	159.5±44.1	-	0.135	0.048	0.985
**HLM start**	158.7±31.2	0.677	182.8±37.7	0.781	128.7±58.3	0.052	0.274	0.003	0.241
**30min**	152.6±27.3	0.114	189.8±23.9	0.994	106.9±47.3	0.0001	0.120	<0.0001	0.038
**VAD start/ 60min**	153.1±22.6	0.734	195.7±31.9	0.999	120.7±45.3	0.009	0.016	<0.0001	0.182
**20min/90min**	160.6±20.4	0.999	191.8±27.5	0.999	131.3±49.6	0.090	0.224	<0.001	0.256
**40min/120min**	160.5±23.4	0.999	189.9±24.7	0.994	146.1±44.7	0.814	0.263	0.023	0.720
**60min/150min**	163.9±23.4	0.971	185.6±29.1	0.895	125.4±50.3	0.364	0.485	<0.001	0.097
**End of OP**	153.6±26.9	0.917	178.9±27.6	0.377	160.4±74.2	0.999	0.372	0.501	0.929
**6h post**	161.6±21.9	0.999	160.4±30.2	0.0002	223.6±69.7	0.021	0.992	<0.001	0.002
**12h post**	154.9±25.3	0.994	150.9±28.1	<0.0001	233.8±62.1	0.005	0.975	<0.0001	<0.0001
**24h post**	152.8±24.6	0.899	158.2±23.9	<0.0001	232.4±61.6	0.004	0.933	0.0001	0.0001
**2-months**	157.7±46.7		164.1±29.7		-		0.999	-	-

vWF:Ac: Von Willebrand Factor activity level; For further abbreviation please refer to Table 3.

**Table 5:** Von Willebrand factor activity/Von Willebrand factor antigen ratio values.

Time points LVAD groups/ CABG group	HVAD (n = 8)	*p*-values within HVAD group	HM II (n = 12)	*p*-values within HMII group	CABG (n = 13)	*p*-values within CABG	*p*-values HMII vs. HVAD	*p*-values HMII vs. CABG	*p*-values HVAD vs. CABG
	vWF:Ac/ vWF:Ag	compared to baseline	vWF:Ac/ vWF:Ag	compared to baseline	vWF:Ac/ vWF:Ag	compared to baseline			
**Baseline**	0.57±0.11	**-**	0.87±0.23	**-**	0.95±0.30	-	**0.019**	0.683	**0.0016**
**HLM start**	0.58±0.11	0.999	0.92±0.20	0.870	0.99±0.31	0.948	**0.0061**	0.779	**0.0006**
**30min**	0.69±0.14	**0.002**	0.95±0.19	0.086	1.01±0.24	0.992	**0.044**	0.897	**0.013**
**VAD start/ 60min**	0.60±0.11	0.303	0.92±0.24	0.762	1.01±0.31	0.930	**0.013**	0.584	**0.0006**
**20min/ 90min**	0.63±0.12	0.666	0.90±0.23	0.987	1.01±0.23	0.944	**0.041**	0.484	**0.0016**
**40min/ 120min**	0.61±0.08	0.849	0.90±0.20	0.989	1.09±0.33	0.195	**0.025**	0.137	**<0.0001**
**60min/ 150min**	0.62±0.11	0.810	0.88±0.25	0.999	0.99±0.47	0.999	**0.042**	0.541	**0.002**
**End of OP**	0.62±0.12	0.968	0.79±0.23	0.521	1.09±0.29	0.081	0.265	**0.007**	**<0.0001**
**6h post**	0.57±0.06	0.999	0.68±0.19	0.060	1.05±0.30	0.705	0.577	**0.0005**	**<0.0001**
**12h post**	0.58±0.08	0.999	0.65±0.20	**0.029**	0.91±0.23	0.991	0.776	**0.021**	**0.006**
**24h post**	0.59±0.07	0.999	0.64±0.17	**0.013**	0.92±0.25	0.999	0.868	**0.012**	**0.006**
**2-months**	0.77±0.13	**0.005**	0.80±0.13	0.955	-		0.999	-	-

vWF:Ac/vWF:Ag: Von Willebrand Factor activity/ von Willebrand antigen ratio; For further abbreviation please refer to Table 3.

#### HMII group (n = 12)

HMII patients pre-operatively presented with a high vWF:Ag (237.6 ± 47.9%) which was above the normal range and decreased significantly between HLM start and VAD start and two months postoperatively (186.3±43.3%, *p* = 0.0107). All postoperative values at 6hr, 12hr and 24hr were significantly higher compared to the 2-month postop value. Comparing the baseline value of vWF:Ag to all other peri-operative values no significant changes were detected.

The vWF:Ac mean value at baseline (198.6 ± 24.7%) was also above the normal range. The vWF:Ac decreased significantly beginning at 6hr postoperatively and continued its decrease until two months postop (Table 3).

HMII patients presented with normal baseline vWF:ratio (0.87 ± 0.23) which decreased significantly during the postoperative period. Beginning at the 6hr postoperative point, this significant decrease continued until 24hr postoperatively (0.64±0.17) and values normalized after two months (0.80 ± 0.13, Table 5). Despite that, we could detect a loss of HMWM of vWF via multimer analysis two months after surgery in all patients from HMII group (one patient died during the first 60 days after implantation). However, the limited comparability of 2-months to perioperative data based on two different diagnostic assays should be considered.

#### HVAD group (n = 8)

The baseline vWF:Ag mean value (278.1±46.6%) was above the normal range in the HVAD group. The perioperative values did decrease, but despite the time point 30min. after HLM start, not significantly compared to the baseline value. The 2-months postoperative value was significantly lower than the baseline value (206.4±26.9% vs. 278.1±46.6%, *p* = 0.0042). The baseline vWF:Ac mean value (162.5±29.2%) was in the normal range, no significant changes were observed over time. Patients who were chosen for HVAD implantation already presented preoperatively with a decreased vWF:ratio, indicating a preexisting AvWS. The mean baseline vWF:ratio (0.57±0.11) was below the normal range. Changes during peri- and early postoperative period were not significant compared to the baseline values for both vWF:Ac and vWF:ratio (despite 30min. after HLM start time point). The vWF:ratio recovered significantly and gained low normal levels two months after HVAD implantation (0.77±0.13 vs. 0.57±0.11, *p* = 0.0056). Nevertheless, we detected a loss of HMWM of vWF via multimer analysis two months after implantation in all HVAD patients (2 patients died during the first 60 POD).

#### CABG group (n = 13)

The baseline vWF:Ag mean value (178.8±64.6%) was above the normal range.

The vWF:Ag decreased significantly perioperatively starting with HLM-start and continued for 150 min. The vWF:Ag then significantly increased postoperatively compared to the baseline value (baseline vs. 12hr: 178.8±64.6% vs. 261.5±56.5%, *p* = 0.0005; baseline vs. 24hr: 178.8±64.6% vs. 258.6±54.2%, *p* = 0.0006). The baseline vWF:Ac mean value (159.5±44.1%) was in the normal range. The vWF:Ac decreased significantly during the early perioperative phase (baseline vs. HLM+30min:159.5±44.1% vs.106.9±47.3%, *p* = 0.0001; baseline vs. 60 min: 159.5±44.1% vs. 120.7±45.3%, *p* = 0.009), was normalized during the rest of the operation, then significantly increased again postoperatively.

The vWF:ratio was in the normal range at any time point. We did not detect any significant changes in the ratio neither peri- nor postoperatively.

### vWF profile between groups

vWF:Ag and vWF:Ac levels and vWF:ratios are presented in Tables 3–5 and Fig 2.

#### HMII vs. HVAD

The vWF:Ag did not differ significantly at any measured time point between the two groups HVAD and HMII. The vWF:Ac was significantly lower in HVAD group compared to the HMII group only at time point VAD-start (HVAD vs. HMII: 153.1±22.6% vs. 195.7±31.9%, *p* = 0.016), despite that the vWF:Ac did not differ significantly between the two groups at any other time point. Although, the vWF:ratio did differ between the two groups starting at baseline time point with a ratio significantly lower in the HVAD group. This significant difference vanished at the end of surgery. Postoperatively, there was no significant difference in the vWF:ratio between HVAD and HMII group.

#### HMII vs. CABG

The vWF:Ag levels were significantly higher in the HMII group compared to the CABG group from the baseline until end of surgery. Postoperatively, there was no significant difference in the vWF:Ag levels between the two groups.

The vWF:Ac levels were also significantly higher in the HMII group from the baseline until 60 min after VAD-start/150 min. after HLM-start. Postoperatively, the vWF:Ac levels of HMII group were significantly lower than CABG levels. At the baseline and during the operation, the vWF:ratio did not differ significantly between the two groups. The HMII patients had a significantly lower vWF:ratio at end of surgery and at all postoperatively measured time points than the CABG patients.

#### HVAD vs. CABG

Patients in the HVAD group had significantly higher vWF:Ag levels than patients in CABG group from the baseline until end of the surgery. Postoperatively, the vWF:Ag did not differ between groups significantly. The vWF:Ac level did not differ between the two groups from baseline to end of surgery, despite HLM+30 time-point, when vWF:Ac level was significantly lower in the CABG group. Postoperatively, the vWF:Ac levels were significantly higher in the CABG group compared to the HVAD group. The vWF:ratio was significantly lower in the HVAD group in all measured time points.

### Bleeding events and blood transfusion products

The data for bleeding events and blood transfusion products are presented in Table 2. The bleeding events per patient-years did not differ between the two groups HVAD and HMII (HMII vs. HVAD: 0.67 vs. 0.85, *p* = 0.685). Early bleeding events occurred at a median of 6±5 POD. 4 patients of each group had an early bleeding event: 1 patient in the HMII group and 3 patients in the HVAD group had pericardial tamponade; 3 patients in the HMII group and 1 patient in the HVAD had hemothorax, all patients were re-operated (Table 2). Also, late bleeding events per patient-years did not differ between the CF-LVAD groups (HMII vs. HVAD: 0.09 vs. 0.28, *p* = 0.785). One patient in the HMII group had two episodes of GI bleeding at 78 and 100 POD; the same patient had epistaxis requiring intervention and the transfusion of packed red blood cells (PRBC) at 123 POD. Two patients in the HVAD group had three episodes of GI bleeding, at 53, 71 and 83 POD.

During the CF-LVAD implantation, the need of PRBCs and platelet concentrates (PCs) transfusion did not differ between the two groups HVAD and HMII. Patients receiving HMII required significantly more fresh frozen plasma (FFPs) transfusions during surgery than HVAD patients. HMII patients required significantly more PRBCs, FFPs and PCs transfusion during surgery compared to CABG group (HMII vs. CABG: 2.9±1.7 vs. 1.4±1.5, *p* = 0.028 and 3.2±1.8 vs. 0.7±2.3, *p* = 0.007 and 1.7±1.2 vs. 0.4±0.6, *p* = 0.013).

The need of postoperative blood products transfusion did not differ between the two CF-LVADs groups HVAD and HMII. Both CF-LVADs groups had significantly more postoperative PRBCs and FFPs transfusions than CABG patients (HVAD vs. CABG: 4.7±4.5 vs. 0.6±0.9,*p* = 0.004 and 1.7±3.9 vs. 0.0; HMII vs. CABG: 4.3±4.0 vs. 0.6±0.9, *p* = 0.003 and 1.5±1.9 vs. 0.0).

After performing Pearson’s correlation test, we could not detect any linear correlation between the vWF:ratio and the need of blood products transfusion, at any measured time point neither during surgery nor postoperative.

**Fig 3:**
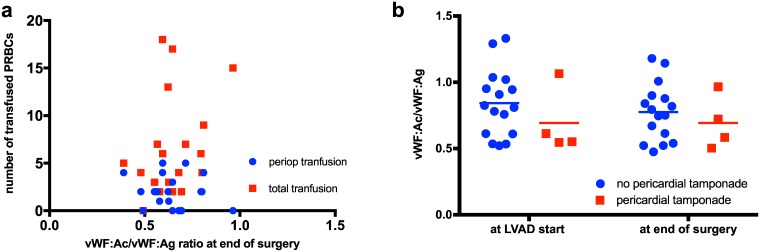
Correlation between vWF:ratio, postoperative pericardial tamponade and blood product transfusion. a) Demonstrates no correlation between vWF:ratio at end of surgery and the need of RPBC transfusion, b) Demonstrates the correlation between vWF:ratio at LVAD start and at end of surgery with the incidence of postoperative pericardial tamponade. LVAD: left ventricular assist device, PRBC: packed red blood cell.

We could detect a correlation between vWF:ratio < 0.7 and the incidence of pericardial tamponade at time point VAD-start (*r* = -0.583, *p* = 0.007) and a correlation between vWF:ratio <0.7 with pericardial tamponade at the end of surgery (*r* = -0.461, *p* = 0.035) (Fig 3).

## Discussion

Tiede *et al*. [15] showed that the combined interpretation of vWF:Ag, vWF:Ac and vWF:Rco/vWF:Ag = vWF:Ac/vWF:Ag increases the sensitivity of AvWS diagnosis to a maximum of 86%. Additionally, the vWF multimer analysis is still an important diagnostic method in detecting AvWS [15, 16], especially for the detection of structural abnormalities [17]. In a previous study, we demonstrated a very strong correlation between the selected method to measure vWF:Ac, vWF:Ag and its ratio with the loss of HMWM detected by electrophoresis and western blot [11]. These findings are in agreement with other clinical AvWS studies: A study by Vincentelli *et al*. [18] analyzed AvWS in patients with moderate or severe aortic stenosis, and they found a good correlation between vWF:RCo/vWF:Ag with the loss of HMWM only in patients with severe aortic stenosis, while patients with moderate aortic stenosis still had a ratio above 0.7 while loss of HMWM still could be detected via multimer analysis. According to our results from the previous in-vitro study and to the findings from Vincentelli *et al*. and Tiede [11, 15, 18] findings, multimer analyses are highly sensitive for predicting of vWS even when only slight changes in VWF are presented, but for patients with moderate to severe degradation of vWF, the vWF:RCo/vWF:Ag or vWF:AC/vWF:Ag correlated well with the loss of HMWM, which applies to the patients with high risk for bleeding. In our study, we now used this knowledge to detect the perioperative onset of AvWS with the limitation, that little developed AvWS might not reliably be detected.

In the last years, the loss of HMWM of vWF after CF-LVAD implantation has gained large attention as a presumable cause for bleeding after CF-LVAD implantation, very similar to Heyde syndrome described in patients with severe aortic stenosis [19]. High shear stress changes the three-dimensional structure and enhances proteolysis of the vWF by ADAMTS-13 [4, 20]. The loss of HMWM of vWF leads to the loss of the ability to bridge the binding of platelets to collagen resulting in disturbed hemostasis.

Geisen *et al*. [21] first reported loss of HMWM of vWF in patients supported with CF-LVADs (HMII) and pulsatile VADs despite comparable vWF:Ag values to heart transplantation (HTX) recipients. Many previous studies [7–10] demonstrated the onset of AvWS in CF-LVADs in the early postoperative period, yet the earliest time window monitored was 24 hours after surgery by Malehsa *et al*. [10].

In our study, we proved that the onset of AvWS is either a very early postoperative phenomenon that occurs immediately at the end of surgery or is already present before surgery. In addition, our results show that at the end of surgery the vWF:Ac/vWF:Ag ratio was significantly lower in both CF-LVAD groups compared to the control group. In accordance with Meyer *et al*. [22], we could not detect any significant differences in the postoperative vWF profile while comparing an axial pump (HMII) to a centrifugal pump (HVAD). Although, there were perioperative differences between both pump types with regards to AvWS development, which are most likely due to significant differences in patients’ baseline vWF:ratios. It seems like a patient’s already diminished preoperative vWF:ratio could not be diminished further by LVAD. The important finding that a subgroup of end-stage heart failure patients already had a very low preoperative vWF:Ac/vWF:Ag ratio<0.7, indicating the onset of AvWS even before CF-LVAD implantation, confirms the divers results of other groups: Heilmann *et al*. [8] and Crow *et al*. [23] could also identify AvWS in some LVAD patients prior to surgery. There are several studies and reviews examining the pathophysiology of AvWS: Michiels et al. [24] gives a good review about vWF for congenital and acquired vWS, including aortic stenosis, cancer or autoimmune pathologies. Furthermore, strong correlations between vWF levels and *Helicobacter pylori*, C-reactive protein (CRP) and age [22, 25] were reported in literature. We examined these parameters in our patient cohort with the following results: None of these patients had a severe aortic stenosis or other pathological findings, which could explain the low vWF:Ac/vWF:Ag ratio preoperatively. *Helicobacter pylori* were not analyzed preoperatively in any patient. All patients had normal CRP values preoperatively; otherwise the surgery was postponed until normalized CRP values were presented. However, the subgroup of patients who had a low vWF:Ac/vWF:Ag ratio were older compared to the rest of patients, who underwent CF-LVAD implantation.

In our study, both CF-LVADs groups presented with supra-normal baseline vWF:Ag, significantly different to our control group. We believe this is due to the severity of heart failure of these kind of patients (LVAD candidates, NYHA III-IV) and which is in accordance with results of Chin *et al*. [26] showing abnormal high vWF:Ag levels in patients with decompensated (NYHA III-IV) and acute heart failure compared to control group.

Control patients who underwent routine CABG surgery experienced a drop in both levels of vWF:Ag and vWF:Ac during surgery, which might be due to dilution effects of HLM, but recovered immediately after surgery to reach supernormal values compared to the baseline, possibly due to an HLM-associated acute phase reaction. This immediate recovery of both vWF:Ag and vWF:Ac is possibly due to increased endothelial and thrombocyte-derived release of vWF, an acute-phase response of vWF as described by Lippi *et al*. and Pottinger *et al*. [27, 28], as well as due to postoperative diuretic effects. We could not detect an AvWS generating effect of the HLM on the vWF profile.

### Bleeding events and blood transfusion

In our study, early bleeding events (hemothorax and pericardial tamponade) with required re-exploration, occurred in 50% of HVAD patients and in 33.3% of HMII patients. Our findings about the incidence of early bleeding events in CF-LVAD patients are consistent, although higher, with results from various groups: Genovese *et al*. [29] found an incidence of 25% of re-operation due to early bleeding in 195 HMII patients. John *et al*. [30] observed early bleeding and re-exploration in 16% of HMII patients. Slaughter *et al*. and Aaronson *et al*. [31, 32] found early bleeding and re-operation in only 14.8% and 14.3% respectively of HVAD patients. Our finding about the incidence of GI bleeding during the monitored period in both groups HVAD (25%) and HMII (8.3%) are in accordance with many previous studies [2, 31–34]. We could not detect any significant difference in any bleeding rates between the two groups HVAD and HMII; these findings are consistent with Stulak *et al*. [35].

In accordance with Goda *et al*. [7], we could not detect linear correlation between vWF levels with the amount of blood products being transfused peri- and postoperatively. Also in accordance with Goda *et al*. and Meyer *et al*. [7, 22], we found no correlation between low postoperative vWF:ratio and incidence of postoperative pericardial tamponade with required re-operation. But we found a correlation between the vWF:ratio < 0.6 at early perioperative time points (VAD-start and end of surgery) with the incidence of postoperative pericardial tamponade with required re-operation.

### Perspectives

INNOVANCE^®^ Ac kit is a fast and easy to use method for screening of AvWS in heart failure patients undergoing cardiac surgery. Identifying patients with AvWS before surgery will give surgeons the opportunity to act early and start with prophylactic procedures to avoid bleeding events, such as administration of Desmopressin (1-desamino-8-D-arginine vasopressin) [36, 37] or vWF concentrate replacement with Haemate-P^®^ for surgeries and bleeding events [38, 39].

### Limitations of the study

Our data suffers the usual shortcomings of a small-cohort single-center non-randomized study. A higher patient number enrolled in a multicenter trial would be preferable. Lack of randomization might potentially lead to selection bias. In this study, the selection of whether patients were to receive HMII or HVAD was made by the cardiac-surgeon team independently of this study protocol. Although HVAD and HMII cohorts only differed in age and BMI, patients receiving an HVAD had a significant lower preoperative vWF:ratio indicating preoperative AvWS. Neither the surgeon nor the study team had had information about the baseline vWF profile before or during surgery. But this fact may limit generalizability of the results.

Another limitation of this study is the absence of a continuous perioperative vWF multimer analysis as a still used standard. The vWF multimer analysis is a costly and time-consuming analysis, which makes it an unsuitable method to be clinically used for perioperative screening. As different groups already demonstrated a correlation between the selected method of measuring vWF:ratio to the vWF multimer analysis in previous studies [15, 17, 18](see discussion section), we did not further follow this method, accepting that little developed AvWS may not be detected.

## Conclusion

As a leadoff group, we investigated *perioperative* vWF profiles in LVAD patients in frequent intervals prior to and during the surgery as well as in the postoperative time frame. We compared vWF profiles and bleeding events in two CF-LVAD cohorts (HVAD, HMII) and patients undergoing CABG procedure, acting as a control group. We could find no incidence of AvWS amongst the control group over the investigated timeframe. The presumably dilutive effect of the heart lung machine on vWF:Ag level vanishes immediately at the end of surgery, possibly as part of an acute-phase response. In our LVAD cohorts, we found a subgroup of patients, who already suffered preoperative AvWS. In this patient cohort we could show, that the preoperative diminished vWF was not diminished even more by CF-LVAD implant. In the CF-LVAD group without preoperative AvWS the onset of AvWS begins immediately after the end of surgery, at this time there was no more difference between LVAD cohorts. A vWF:ratio < 0.7 at VAD start or end of surgery was found to be a risk factor in LVAD patients correlating to higher incidences of pericardial tamponade and re-operation. Our results show that there are still open questions to address with regard to LVAD-related AvWS since we think that vWF contributes to the complex puzzle of bleeding and thrombosis complications in LVAD patients.

## Supporting information

S1 File Include all dataset of pre-, peri- and postoperative information. (XLSX)

S2 File All vWF:Ag measurments. (CSV)

S3 File All vWF:Ac measurmnets. (CSV)

S4 File All vWF:ratio measurments. (CSV)
